# Potential protein blood-based biomarkers for cognitive dysfunction in Parkinson’s disease: a systematic review and network meta-analysis

**DOI:** 10.3389/fnagi.2026.1724548

**Published:** 2026-02-10

**Authors:** Youxue Tu, Changmei Lu, Bao Wu, Yuping Yang, Jiapeng Li, Qingping Su, Zhenhua Zhao

**Affiliations:** 1 Department of Rehabilitation, Fuzhou First General Hospital Affiliated with Fujian Medical University, Fuzhou, China; 2 Department of Rehabilitation, Fujian University of Traditional Chinese Medicine, Fuzhou, China; 3 The Institution of Rehabilitation Industry, Fujian University of Traditional Chinese Medicine, Fuzhou, China; 4 Department of Rehabilitation, The Fifth Hospital of Xiamen, Xiamen, China; 5 Shengli Clinical Medical College of Fujian Medical University, Fuzhou, China; 6 Rehabilitation Medicine Center, Fuzhou University Affiliated Provincial Hospital, Fuzhou, China; 7 Department of Neurology, Fuzhou University Affiliated Provincial Hospital, Fuzhou, China

**Keywords:** cognitive dysfunction, mild cognitive impairment, network meta-analysis, Parkinson’s disease, protein biomarkers

## Abstract

**Objectives:**

Parkinson’s disease (PD) patients frequently develop mild cognitive impairment (PD-MCI) and may progress to Parkinson’s disease dementia (PDD). Despite progress in understanding PD pathophysiology, reliable blood-based biomarkers for early detection and monitoring of cognitive decline remain lacking. This study aims to systematically evaluate and compare blood-based protein biomarkers for cognitive impairment in PD through a network meta-analysis (NMA).

**Methods:**

A systematic search was conducted across PubMed, Embase, Web of Science, Scopus, and the Cochrane Library from database inception to January 23, 2025, to identify studies investigating blood protein biomarkers for cognitive impairment in PD patients. Studies comparing biomarker levels between PD patients with cognitive impairment (PD-CI) and those with normal cognition (PD-NC) were included. Two independent reviewers extracted data, and risk of bias was assessed using the Newcastle-Ottawa Scale (NOS). A frequentist NMA was performed using Stata 15.0 with a random-effects model to estimate standardized mean differences (SMD) and 95% confidence intervals (CI).

**Results:**

A total of 47 studies were included, encompassing biomarkers from five functional categories: metabolic function, neuronal function, inflammatory and immune functions, blood and vascular functions, and others. Key findings demonstrated significant alterations in several biomarkers in PD-CI compared with PD-NC. Cystatin C (Cys C) levels were significantly elevated in PD-CI (SMD = 0.81, 95% CI: 0.32, 1.30). Glial cell line-derived neurotrophic factor (GDNF) levels were significantly reduced in PD-CI (SMD = −1.06, 95% CI: −1.71, −0.41). Neurofilament light chain (NfL) levels were significantly elevated in PD-CI (SMD = 0.72, 95% CI: 0.39, 1.05). Interleukin-6 (IL-6) levels were significantly higher in PD-CI (SMD = 0.20, 95% CI: 0.01, 0.92).

**Conclusion:**

This NMA identifies Cys C, GDNF, NfL, and IL-6 as promising blood-based biomarkers for detecting cognitive impairment in PD. These biomarkers reflect diverse pathological processes and hold potential for facilitating early diagnosis and monitoring, thereby improving patient management. Further research is warranted to validate these findings and explore their clinical utility.

**Systematic review registration:**

https://www.crd.york.ac.uk/PROSPERO/view/CRD42023488801, Identifier: CRD42023488801.

## 1 Introduction

Parkinson’s disease (PD) is a progressive neurodegenerative disorder affecting millions worldwide, with cognitive impairment being a debilitating feature (Loo et al., 2025). Up to 80% of PD patients develop mild cognitive impairment (PD-MCI) within 5 years of diagnosis (Yang et al., 2023), and approximately 50% progress to Parkinson’s disease dementia (PDD) over a decade (Hely et al., 2008). This cognitive decline significantly impairs daily functioning and substantially burdens caregivers (Mantovani et al., 2024). Current diagnostic methods, such as neuropsychological assessments, are subjective and time-consuming (Litvan et al., 2012), while neuroimaging and cerebrospinal fluid (CSF) biomarkers are costly and invasive, limiting their widespread use (Parnetti et al., 2019).

Blood-based biomarkers represent a promising, minimally invasive alternative for detecting cognitive impairment in PD (Dennis and Strafella, 2024). However, existing studies have reported inconsistent findings regarding the association between specific biomarkers and cognitive decline, largely due to heterogeneity in study designs and methodologies (Aamodt et al., 2021; Ye et al., 2021). For instance, blood neurofilament light chain (NfL) has been reported to predict subsequent cognitive decline in some longitudinal cohorts, whereas other studies observed weak or non-significant baseline associations with cognition in unstratified PD populations, highlighting heterogeneity in candidate biomarkers (Niemann et al., 2021; Li Q. et al., 2022; Pedersen et al., 2024). Importantly, this study is not only concerned with whether an individual biomarker differs between PD patients with cognitive impairment (PD-CI) and PD patients with normal cognition (PD-NC) but also aims to interpret and compare multiple candidate biomarkers under a consistent statistical framework to inform clinical prioritization and the future design of multi-biomarker panels. In the available literature, the biomarkers assessed are not identical across studies, whereas PD-NC serves as the comparator group in most reports, naturally forming a network structure with PD-NC as the common reference node. Therefore, we applied a network meta-analysis (NMA) framework to synthesize all PD-CI versus PD-NC contrasts within a single pre-specified model and to present effect estimates on a uniform scale, facilitating exploratory comparative interpretation across biomarkers when head-to-head evidence is limited.

This systematic review and NMA aims to: (1) synthesize and quantitatively compare the evidence on blood-based protein biomarkers for PD-CI; (2) categorize these biomarkers into functional domains to elucidate their distinct biological roles (Kim et al., 2022); and (3) identify the most promising biomarkers for clinical translation through a network meta-analytical approach. The findings from this NMA will provide clinicians with a robust evidence base to guide the selection of blood-based biomarkers for early detection and monitoring of cognitive impairment in PD, potentially leading to improved patient outcomes.

## 2 Materials and methods

### 2.1 Literature search and selection criteria

This systematic review and NMA was performed according to the Preferred Reporting Items for Systematic Reviews and Network Meta-Analyses (PRISMA-NMA) guidelines (Hutton et al., 2015). Electronic databases (PubMed, Embase, Web of Science, Scopus, and Cochrane Library) were systematically searched for studies that reported data on blood-based protein biomarkers associated with cognitive dysfunction in patients with Parkinson’s disease (PD) versus controls from database inception to January 23, 2025. The initial study protocol was preregistered in the International Prospective Register of Systematic Reviews (PROSPERO, CRD42023488801). The full search strategy is listed Supplementary Table S1 and additional literature was added by hand-searching references of relevant reviews and meta-analyses.

Original articles were included if they met the following PICOS-based criteria (Population, Intervention or Exposure, Comparator, Outcomes, and Study design): (1) adult participants with a clinical diagnosis of Parkinson disease, with at least two groups defined by cognitive status, including at least one PD-CI group (PD-MCI and/or PDD) and a comparator group of PD-NC; (2) measurement of blood-based protein biomarkers in peripheral blood (serum or plasma) using any validated laboratory assay, with biomarker concentrations reported separately for PD-CI and PD-NC; (3) outcomes including quantitative biomarker concentration data enabling calculation of between-group effect sizes, with cognitive status classified by validated instruments or diagnostic criteria (for example, the Mini-Mental State Examination (MMSE), Montreal Cognitive Assessment (MoCA), Clinical Dementia Rating (CDR), Parkinson’s Disease-Cognitive Rating Scale (PD-CRS), the Diagnostic and Statistical Manual of Mental Disorders, Fourth Edition (DSM-IV), and/or Movement Disorder Society (MDS) criteria) without imposing additional unified cut-off values, and with PD-MCI and PDD pooled as PD-CI and cognitively normal or non-demented PD pooled as PD-NC for the primary synthesis; (4) study designs included cross-sectional, case–control, and cohort studies.

Studies were excluded for the following reasons: (a) measured biomarker concentrations in postmortem samples, animals, or *in vitro*; (b) duplicated samples that overlapped with other studies; and (c) raw data could not be obtained completely. For several publications reported from the same center, we included the publication that had the greatest sample size.

### 2.2 Data extraction and analysis

Two authors (Youxue Tu and Changmei Lu) independently extracted data from the 47 included studies using a pre-designed form, including study characteristics (first author, year, country, design), participant characteristics (sample size, age, sex, cognitive assessment), biomarker information (marker type, specimen, assay), and outcome data (biomarker levels in PD-CI and PD-NC). Disagreements were resolved by discussion or consultation with a third author (Qingping Su).

For quantitative synthesis, each blood-based protein biomarker was treated as a separate node, with PD-NC as the common comparator. We used a frequentist NMA in Stata 15.0 for biomarkers reported in ≥ 3 studies, applying a random-effects consistency model. Effects were expressed as SMD with 95% CI, calculated from group means, standard deviations, and sample sizes. SMD was chosen to account for differences in assay methods and measurement units. Between-study variance (τ^2^) was estimated using restricted maximum likelihood (REML). Heterogeneity was assessed using τ^2^ and I^2^ statistics, and Cochran’s Q was examined for key biomarkers. Network geometry plots were generated with node size proportional to total sample size and edge thickness proportional to the number of contributing studies. Because the network was predominantly star-shaped and lacked closed loops, formal inconsistency assessment and SUCRA-based ranking were not undertaken; biomarker-to-biomarker comparisons were not considered confirmatory and should be interpreted, at most, as exploratory. Forest plots were produced using Stata’s NMA routines.

### 2.3 Quality assessment of studies

To evaluate the quality of studies included in the systematic review, the NOS was employed, utilizing the original version for cohort and case–control studies and an adapted version for cross-sectional studies (Fernandes et al., 2021). The scale assesses three domains: selection of study participants (up to 4 points), comparability of study groups (up to 2 points), and ascertainment of outcomes (up to 3 points), with a maximum total score of 9 points. Studies were classified into four quality levels: excellent (9 points), good (7–8 points), satisfactory (5–6 points), and unsatisfactory (< 5 points). Studies rated as unsatisfactory were excluded from the NMA to ensure the reliability and consistency of the findings. Two authors (Youxue Tu, Qingping Su) independently assessed quality, resolving disagreements through consensus or consultation with Bao Wu. Full NOS scores are provided in Supplementary Table S2.

## 3 Result

### 3.1 Literature search and study selection

**Figure 1:**
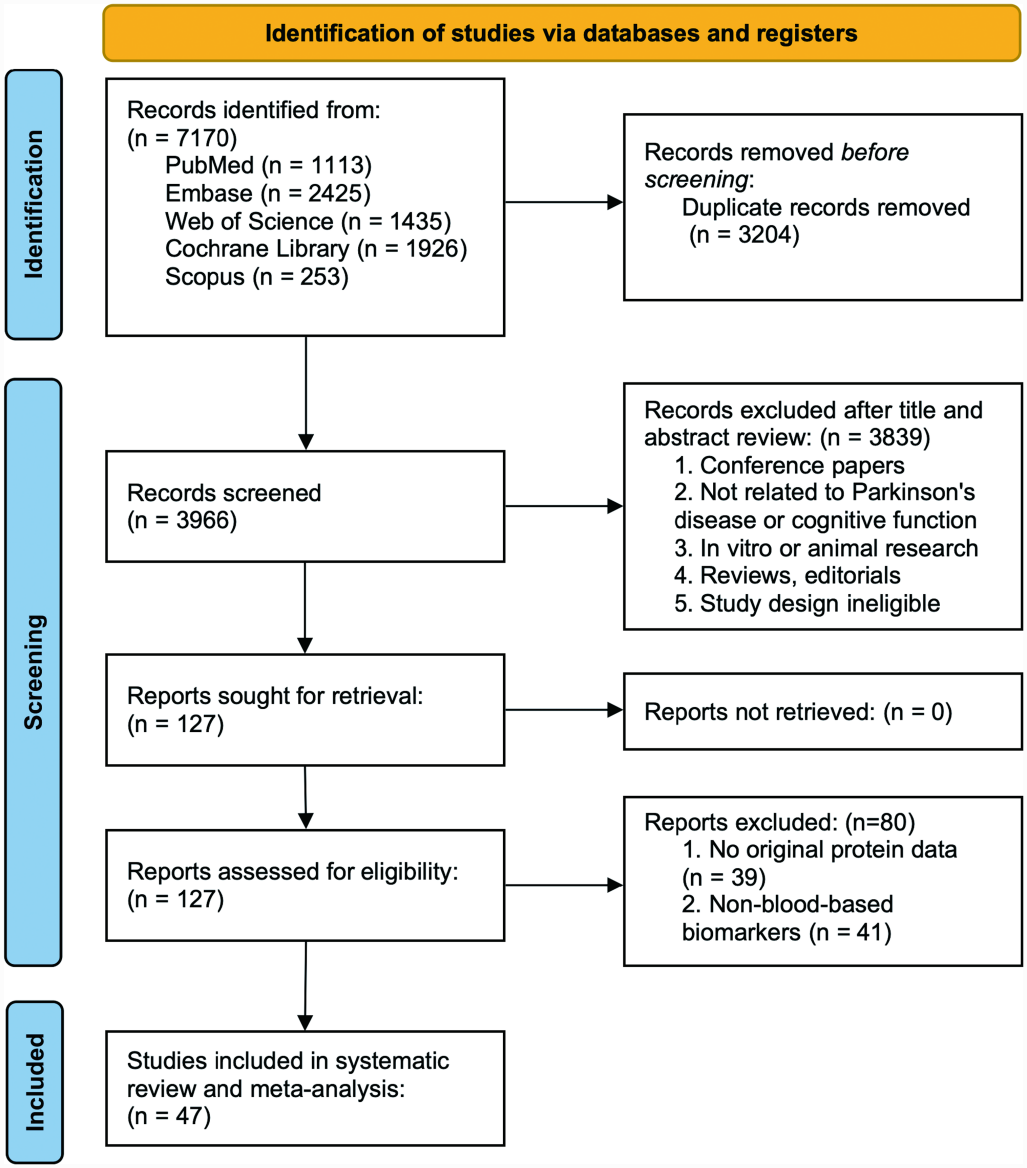
Flow diagram of the study selection.

Our systematic search identified 7,170 records from major databases including PubMed, Embase, Web of Science, Scopus, and the Cochrane Library up to January 2025. After deduplication and screening, we excluded 3,839 irrelevant records and a further 80 studies during full-text review because 39 lacked protein data and 41 analyzed non-blood biomarkers. Ultimately, 47 studies met the inclusion criteria and were included in the network meta-analysis, as summarized in Figure 1.

### 3.2 Characteristics of the included studies

Table 1 shows the selected studies that reported potential blood biomarkers for Cognitive Dysfunction in Parkinson’s Disease. The included studies were published between 1993 and 2025 and were conducted across various countries. These studies typically compared PD patients with cognitive impairment [PD-CI, including PD with mild cognitive impairment (PD-MCI) and PD with dementia (PDD)] to PD patients without cognitive impairment (PD-NC) and/or healthy controls (HC). In some cases, additional comparator groups such as Alzheimer’s disease (AD), dementia with Lewy bodies (DLB), or frontotemporal dementia (FTD) were included. Sample sizes varied widely, ranging from 46 to 928, with male and female participants reported separately in most studies. The mean age of participants generally ranged from the late 50s to the mid-70s, reflecting the typical age profile of PD populations. Cognitive function was assessed using a variety of standardized tools, including the MMSE, MoCA, MDS criteria for PD-MCI or PDD, CDR, and other neuropsychological batteries. Blood specimens analyzed were predominantly serum and plasma, with a few studies using whole blood cell pellets or peripheral blood. Potential blood biomarkers were identified in the selected studies.

**Table 1:** Summary of the 47 selected studies reporting potential biomarkers for cognitive dysfunction in Parkinson’s disease.

Author and year	Country	Study groups	Sample size (M/F)	Age (y)	Outcome measurement Tool	Specimen	Potential blood protein biomarkers
Wu et al. (2024)	China	HC PD-NC* PD-CI*	28/32 28/18 30/24	57 (49, 63) 57 (51, 63) 71 (63, 75)	MOCA	Serum	Cys C, HDL, LDL, sdLDL, ApoA, ApoB, Lpa, SAA, Lp-PLA2
Abd Elhadi et al. (2019)	Israel	PD-ND* PDD* HC	20/12 11/3 19/26	70 ± 6.0 74.1 ± 7.6 69.5 ± 8.3	MOCA	Whole blood cell-pellets	Total (methanol) a-Syn, Total (cyclohexene) a-Syn, PK^res^ a-Syn, PSer129, Oxidized a-Syn, Hemoglobin, Iron, H-ferritin
Hou et al. (2024)	China	Training group PD-CI* PD-NC* Test group PD-CI* PD-NC*	140/64 106/75 75/40 24/26	64.85 ± 9.15 61.61 ± 10.02 65.86 ± 8.87 61.96 ± 8.17	MOCA	Plasma	Ferritin, NSE
Li C. H. et al. (2022)	Taiwan	HC PD-NC* PD-MCI* PDD*	10/10 19/11 19/11 17/10	62.6 ± 8.0 65.2 ± 7.9 70.3 ± 7.2 75.5 ± 8.2	MMSE	plasma	Aβ42, Aβ40, Tau
Li et al. (2021)	China	PD-NC* PD-CI* HC*	108/76 30/36 174/160	59.2 ± 10.68 64.12 ± 8.55 60.76 ± 10.99	MMSE/MOCA	Plasma	pS-*α*-syn-RBC
Mao et al. (2023)	China	PD-NC* PD-MCI* PDD* HC	21/15 21/16 10/20 18/19	59.0 ± 9.3 63.3 ± 7.8 69.2 ± 5.5 62.2 ± 6.2	MOCA, MDS	Serum	NfL, GFAP
Tsai et al. (2021)	Taiwan	PD-NC* PDD* HC	53/43 24/15 13/21	65.8 ± 10.7 72.0 ± 10.8 67.6 ± 7.9	MMSE	Plasma	Aβ42, T-tau, Aβ40, *α*-Syn
Song et al. (2013)	Korea	PD-ND* PDD* HC	38/34 22/23 50/34	69.74 ± 10.32 71.44 ± 13.40 73.18 ± 19.75	MMSE, CDR	Serum	hs-CRP, Fibrinogen
Lin et al. (2018)	Taiwan	AD MCI PD-ND* PDD* HC	56/63 27/29 13/13 14/9 31/28	77.3 ± 5.1 76.0 ± 5.6 69.6 ± 10.8 76.3 ± 9.1 77.0 ± 6.2	MMSE, CDR	Plasma	NfL
Dong et al. (2021)	China	HC PD-ND* PDD*	19/22 18/18 15/17	59.27 ± 12.96 66.21 ± 10.42 70.44 ± 8.89	MMSE	Plasma	HbA1c, ApoA1, ApoB, Lpa
Liu et al. (2022)	China	PDD* PD-ND* HC	17/14 21/26 26/21	71.1 ± 7.0 62.4 ± 8.0 66.4 ± 9.3	MMSE	Plasma	SOD, Cys C, hs-CRP
Dong et al., 2017	China	PD-ND* PDD* HC	36/23 9/8 29/32	68.10 ± 8.98 73.47 ± 7.89 67.87 ± 5.74	MMSE	Serum	Albumin, ALT, AST, BChE activity
Liu et al. (2024)	China	HD PD-NC* PD-MCI*	45 52 53	63.40 ± 8.17 64.45 ± 8.15 64.02 ± 9.70	MMSE, MOCA	Serum	GDNF
Kuiper (1993)	Netherlands	PD-ND* PDD* AD MSA HC	63 15 10 12 21	65 ± 11 73 ± 8 64 ± 8 66 ± 11 61 ± 17	DSM-III-R	Serum	ACT
Wang et al. (2020)	China	PDD (−)* PDD (+)* DMD (−) DMD (+) PD-DMD (−) PD-DMD (+)	175/135 18/13 192/145 27/8 88/96 16/15	65.0 (56.0, 73.0) 69.0 (59.0, 79.0) 61.0 (52.0, 69.0) 68.0 (57.0, 74.0) 73.0 (66.0, 80.0) 79.0 (72.0, 84.0)	MMSE MOCA	Serum	Fibrinogen, hs-CRP, Albumin, Cys C, ALT
Zhu et al. (2021a)	China	PD-NC* PD-MCI* PDD* HC	28/29 25/9 24/15 16/22	62.8 ± 10.5 64.5 ± 10.3 68.7 ± 8.6 64.9 ± 10.6	MMSE	Plasma	NfL
van Kamp et al. (1995)	Netherlands	PD-NC* PDD* HC	37/37 10/6 11/10	64.5 ± 11.7 74.8 ± 7.1 61.5 ± 15.6	NA	Serum	Trf
He et al. (2025)	China	PD-NC* PD-CI*	50/65 32/62	62 ± 9.92 70 ± 9.77	MMSE	Serum	Aβ1–42
Fan et al. (2020)	New Zealand	HC PD-NC* PD-MCI* PDD*	15/8 46/28 52/19 28/5	74.53 ± 6.57 70.91 ± 7.05 72.89 ± 6.40 76.17 ± 5.34	MDS-TF Level II	Plasma	IGF-1, IGFBP-3
Gmitterova et al. (2020)	Germany	DLB PDD* PD-ND* HC	24 17 13 14	69 (45–84) 69 (60–85) 66 (58–83) 61 (52–80)	NA	Serum	Cg A
Lin et al. (2020)	Taiwan	HC MCI AD PD-NC* PD-MCI* PDD* FTD	31/66 16/25 14/21 31/26 20/9 53/34 6/25	64.0 ± 7.8 72.9 ± 7.9 75.2 ± 11.6 62.4 ± 11.2 66.5 ± 11.8 72.8 ± 8.9 60.7 ± 7.1	MMSE, MDS task force criteria	Plasma	Aβ42, Aβ40, T-tau, p-tau181, a-Syn
Huang et al. (2024)	Taiwan	PD-NC* PD-MCI*	15/9 12/13	71.3 ± 7.4 75.6 ± 9.1	CDR-SB	Plasma	a-Syn, T-tau, Aβ42
Caranci et al. (2013)	Italy	PD-NC* PD-CI* HC	27/16 13/13 57/53	NA NA 64.31 ± 9.17	MDS-UPDRS part I subitems	Plasma	a-Syn
Hiraga et al. (2024)	Japan	LR HR PD-NC* PD-CI* DLB	26/11 53/29 22/18 22/22 8/8	63.8 ± 5.2 64.9 ± 7.6 64.4 ± 9.0 72.8 ± 7.8 78.4 ± 5.4	MOCA-J	Plasma	p-tau181, NfL, a-Syn
Martin-Ruiz et al. (2020)	UK	PD-NC* PD-MCI* HC	80 61 54/45	NA NA 68.0 (63.3, 82.2)	MMSE, MOCA, MDS Level 2 criteria	Serum	p21, p16, CRP, TNF-α, IL-6, IL-10, IFN-*γ*
Yalcin et al. (2025)	Turkey	Controls PD-ND* PDD*	48/109 123 34	73 ± 10 70 ± 7 72 ± 11	MMSE	Serum	α-Klotho
Maetzler et al. (2012)	America	PD-ND* PDD* DLB HC	43/34 15/11 14/14 38/34	68 (44–81) 71 (62–84) 74 (50–83) 57 (40–80)	MMSE, DSM-IV	Serum	TTR
Huang et al. (2022)	China	HC PD-NC* PD-MCI* PDD* ET	34/26 102 31 13 43/39	60.5 ± 11.3 NA NA NA 60.6 ± 8.8	MMSE	Serum	NfL
Pagonabarraga et al. (2022)	Barcelona	PD-MCI* PD-NC* HC	22/19 36/32 21/19	72.2 ± 5 65.3 ± 7 66.0 ± 7	PD-CRS, MOCA	Plasma	NfL, p-tau181
Deng et al. (2023)	Singapore	PD-NC* PD-MCI*	60/38 62/46	61.9 (55.4, 67.9) 66.9 (59.9, 70.4)	NA	Serum	wrCRP, ApoA1, ApoB, NfL, T-tau
Aamodt et al. (2021)	USA	PD-NC* PD-MCI* PDD* HC	55/39 27/7 8/0 200	64.85 ± 7.616 69.15 ± 8.374 75.63 ± 5.476 69 (65, 74)	DRS-2	Plasma	NfL
Samat et al. (2017)	Malaysia	PD-NC* PD-MCI*	13/7 16/10	64 (58, 65) 63 (58, 69)	MoCA	Plasma	a-Syn
King et al. (2019)	United Kingdom	Control LewyPro MCI-AD MCI-LB Control ICICLE-P PD-MCI* PD-NC*	16/4 7/14 25/13 34/30 33/11 44/68	75.9 ± 1.6 78.5 ± 1.4 75.6 ± −1.2 69.5 ± 0.8 71.1 ± 0.1 69.5 ± 6.7	MMSE, MDS modified level 2 criteria	Serum	CRP, IFN-γ, IL-10, IL-2, IL-4, IL-6, IL-8, TNF-α
Contaldi et al. (2022)	Italy	PD-NC* PD-MCI* HC	18/10 18/5 42/16	67.39 ± 9.09 68.80 ± 8.48 69.31 ± 8.18	ACE-R	Peripheral blood	Hemoglobin, CRP
Zhu et al. (2024)	China	HC PD-NC* PD-MCI*	11/21 33/36 36/36	60 ± 11.1 62.1 ± 11 66 ± 10	MDS Type I diagnostic criteria, MOCA	Plasma	NfL, GFAP
Tang et al. (2023)	China	HC PD-NC* PD-MCI* PDD*	6/9 31/29 41/22 14/10	61.93 ± 8.56 58.45 ± 8.94 59.6 ± 10.53 63.83 ± 10.61	MMSE, MDS criteria	Plasma	GFAP, NfL, Tau, p-tau181
Lin et al. (2019)	Taiwan	HC PD-NC* PD-MCI* PDD*	68 51 35 36	68.3 ± 9.3 64.3 ± 10.9 70.3 ± 6.4 79.9 ± 8.3	MMSE, CDR, MDS criteria	Plasma	Total α-synuclein, PSer129
Shi et al. (2021)	China	HC PD-NC* PD-CI*	14/12 13/13 10/17	64.73 ± 3.75 65.04 ± 10.55 68.07 ± 6.81	MMSE, MoCA, CDR	Serum	GDNF, α-pro-GDNF, β-pro-GDNF
Zhu et al. (2021b)	China	PD-NC* PD-CI* HC	22/18 9/9 55/36	68.6 ± 9.2 71.6 ± 10.6 68.0 ± 10.3	MMSE	Serum	ALT, AST, SIRT1
Choj (2016)	Korea	PD-NC* PD-MCI* PDD*	19/29 21/20 11/13	67.2 ± 7.8 70.5 ± 6.0 71.9 ± 6.6	MMSE, MDS criteria	Serum	hs-CRP
Tong et al. (2023)	China	PD-NC* PD-MCI* PDD* HC	27/17 23/18 6/14 27/18	61.66 ± 8.31 65.02 ± 8.61 67.75 ± 6.16 64.56 ± 8.08	MMSE, MDS criteria	Serum	GDNF
Xiong et al. (2022)	China	PD-NC* PD-CI*	5/9 17/9	60.2 ± 8.3 70.7 ± 6.7	MOCA	Serum	LCN-2
Liang et al. (2024)	China	HC PD-NC* PD-MCI* PDD*	21/21 9/9 7/7 7/8	56.88 ± 6.73 58.83 ± 6.90 60.43 ± 8.36 59.60 ± 7.60	MOCA, MDS criteria	Serum	HMGB1
Chiu et al. (2021)	Taiwan	HC AD ADMCI ADD PD-NC* PD-IC* FTD	10/31 17/45 6/30 11/15 11/6 7/6 7/18	65.1 ± 6.8 74.4 ± 7.8 72.7 ± 7.8 76.7 ± 7.5 66.3 ± 13.1 69.8 ± 9.5 63.8 ± 7.4	MMSE, MDS criteria	Plasma	Aβ1–42, p-tau181
Baek et al. (2021)	Korea	HC PD-NC* PD-CI*	115/67 204/107 60/25	60.6 ± 11.5 60.91 ± 9.7 64.5 ± 9.5	MOCA	Serum	NfL
Vesely et al. (2019)	Nitra	PD-NC* PD-MCI*	25/15 9/9	62.8 ± 7.9 66.8 ± 8.2	MMSE, Level 1 MDS Criteria	Serum	C3, C4, IL-6
Sobhani et al. (2018)	Iran	PDD* PD-NC*	6/14 33/22	70.25 ± 9.04 61.03 ± 10.23	MMSEDSM–IV	Serum	hs-CRP, IL-6, ICAM-1, VCAM-1

α-Klotho, alpha klotho; a-Syn, alpha-synuclein; ACE-R, Addenbrooke’s Cognitive Examination-Revised; ACT, α1-antichymotrypsin; AD, Alzheimer’s disease; ADD, Alzheimer’s disease dementia; ALT, alanine transaminase; ADMCI, amnestic mild cognitive impairment due to AD; ApoA, apolipoprotein A; ApoA1, apolipoprotein A1; ApoB, apolipoprotein B; Aβ40, amyloid β40; Aβ42, amyloid β42; Aβ1–42, amyloid-β1-42; AST, aspartate aminotransferase; BChE activity, butyrylcholinesterase activity; CDR, clinical dementia rate; CDR-SB, Clinical Dementia Rating-Sum of Boxes; Cg A, Chromogranin A; CRP, C-reactive protein; Cys C, cystatin C; DLB, dementia with Lewy bodies; DMD (−), type 2 diabetes mellitus without dementia; DMD (+), type 2 diabetes mellitus with dementia; DRS-2, Mattis Dementia Rating Scale; ET, Essential tremor; FTD, frontotemporal dementia; GDNF, glial cell line-derived neurotrophic factor; GFAP, Glial fibrillary acidic protein; HbA1c, hemoglobin A1c; HC, healthy controls; HDL, high-density lipoprotein; HMGB1, High mobility group box-1; hs-CRP, high sensitivity C-reactive protein; HR, High-risk; ICAM-1, soluble inter-cellular adhesion molecule; ICICLE-PD, Incidence of Cognitive Impairment in Cohorts with Longitudinal Evaluation in Parkinson’s Disease; IFN-γ, interferon gamma; IGF-1, Insulin-like growth factor-1; IGFBP-3, Insulin-like growth factor bindingproteins-3; IL-2, interleukin-2; IL-4, interleukin-4; IL-6, interleukin-6; IL-8, interleukin-8; IL-10, interleukin-10; LCN2, Lipocalin-2; LDL, low-density lipoprotein; Lpa, Lipoprotein (a); Lp-PLA2, lipoprotein-associated phospholipase A2; LR, Low-risk; MCI, mild cognitive impairment; MDS, Movement Disorders Society; MCI-AD, patients with prodromal Alzheimer disease; MCI-LB, prodromal Lewy body disease; MDS-TF Level II, movement disorder society-task force; MDS-UPDRS, Movement Disorder Society-Unified Parkinson’s Disease Rating Scale; MMSE, Mini-Mental State Examination; MOCA, Montreal Cognitive Assessment; MoCA-J, the Japanese version of the Montreal Cognitive Assessment; MSA, Multiple System Atrophy; NfL, neurofilament light chain; NSE, neuron-specific enolase; Oxidized a-Syn, Oxidized a-Synuclein; PD-CI, cognitive impairment in Parkinson’s disease; PD-CRS, Parkinson’s Disease – Cognitive Rating Scale; PDD, PD patients with dementia; PDD (−), Parkinson’s disease without dementia; PDD (+), Parkinson’s disease with dementia; PD-DM, Parkinson’s disease with type 2 diabetes mellitus; PD-DMD (−), PD-DM without dementia; PD-DMD (+), PD-DM with dementia; PD-IC, Parkinson’s disease with impaired cognition including Parkinson’s disease dementia and Parkinson’s disease mild cognitive impairment; PD-MCI, PD patients with mild cognitive impairment; PD-NC, Parkinson’s disease with normal cognition; PD-ND, PD with non-dementia; PK^res^ a-Syn, proteinase-K resistant a-Synuclein; pS-α-syn-RBC, Serine 129-phosphorylated alpha-synuclein red blood cell; PSer129, phospho-Serine 129 a-Synuclein; p-tau181, Phospho-tau 181; SAA, serum amylase A; sdLDL, small dense low-density lipoprotein; SIRT1, silent information regulator 1; SOD, superoxide dismutase; TNF-α, tumor necrosis factor alpha; Trf, Transferrin; Total (methanol) a-Syn, Total (methanol) a-Synuclein; Total (cyclohexene) a-Syn, Total (cyclohexene) a-Synuclein; T-tau, total tau; TTR, transthyretin; VCAM-1, soluble vascular cell adhesion; wrCRP, wide-range C-reactive protein.*Indicates that the patient groups marked with it were included in our meta-analysis.

### 3.3 Classification of potential blood biomarkers for cognitive dysfunction in Parkinson’s disease

To facilitate biological interpretation, we adopted a functional classification framework commonly used in reviews of biomarkers for cognitive impairment and neurodegenerative disorders (Kim et al., 2022; Trombetta et al., 2018). Specifically, biomarkers were mapped to major pathophysiological domains implicated in PD-related cognitive decline, including metabolic dysregulation, neuronal injury/protein aggregation, neuroinflammation/immune dysregulation, and blood–vascular or hematologic alterations; remaining pathways were grouped as “others” (e.g., cellular senescence/cell-cycle–related processes). Because several proteins may have pleiotropic biological functions (e.g., cystatin C), assignment was based on the function most frequently discussed in relation to PD-CI in the source articles and supporting reviews, and multifunctional markers are noted in the table footnotes.

Based on this framework, blood-based protein biomarkers for cognitive dysfunction in Parkinson’s disease were classified into five functional categories: metabolic function, neuronal function, inflammatory and immune functions, blood and vascular functions, and others. These categories reflect their biological roles and alterations in PD patients with cognitive impairment (PD-CI) versus those without (PD-NC), as summarized in Table 2 (changes: increase, decrease, no change, or unclear).

**Table 2:** Changes in potential blood-based biomarkers for cognitive dysfunction in Parkinson’s disease.

Category	Level	Potential blood protein biomarkers
Metabolic function	Increase	Cys C
	Decrease	ApoB, HDL
	No change	Albumin, ALT, AST, ApoA, ApoA1, ApoB, HbA1c, LDL, Lpa, sdLDL, Trf, Cys C
	Unclear	ApoA1, ApoB
Neuronal function	Increase	Aβ1–42, Aβ40, CgA, GFAP, NfL, NSE, p-tau181, T-tau, α-Syn
	Decrease	GDNF, pS-α-syn-RBC, SIRT1, Total (methanol) a-Syn, α-Klotho, BChE activity, PK^res^ α-Syn
	No change	Aβ1–42, Aβ40, Aβ42, NfL, PSer129, p-tau181, Tau, T-tau, Total α-synuclein, TTR, a-Syn, Total (cyclohexene) a-Syn, Oxidized a-Syn, α-pro-GDNF, β-pro-GDNF
	Unclear	NfL, T-tau
Inflammatory and immune functions	Increase	C3, CRP, HMGB1, hs-CRP, IL-6, Lp-PLA2
	Decrease	IL-8
	Nochange	C4, CRP, hs-CRP, IFN-γ, IL-10, IL-2, IL-4, IL-6, LCN2, SOD, TNF-α, ACT
	Unclear	wrCRP
Blood and vascular functions	Increase	VCAM-1, Fibrinogen
	No change	ICAM-1, IGF-1, IGFBP-3, Fibrinogen, Hemoglobin
Others	Decrease	p21
	No change	p16, Ferritin, H-ferritin, Iron, SAA

α-Klotho, alpha klotho; a-Syn, alpha-synuclein; Aβ40, amyloid β40; Aβ42, amyloid β42; Aβ1–42, amyloid-β1-42; ACT, α1-antichymotrypsin; ALT, alanine transaminase; ApoA, apolipoprotein A; ApoA1, apolipoprotein A1; ApoB, apolipoprotein B; AST, aspartate aminotransferase; BChE activity, butyrylcholinesterase activity; Cg A, Chromogranin A; CRP, C-reactive protein; Cys C, cystatin C; GDNF, glial cell line-derived neurotrophic factor; GFAP, glial fibrillary acidic protein; HbA1c, hemoglobin A1c; HDL, high-density lipoprotein; HMGB1, High mobility group box-1; hs-CRP, high sensitivity C-reactive protein; ICAM-1, soluble inter-cellular adhesion molecule, IFN-g, interferon gamma; IGF-1, Insulin-like growth factor-1; IGFBP-3, Insulin-like growth factor binding proteins-3; IL-2, interleukin-2; IL-4, interleukin-4; IL-6, interleukin-6; IL-8, interleukin-8; IL-10, interleukin-10; LCN2, Lipocalin-2; LDL, low-density lipoprotein; Lpa, Lipoprotein (a); Lp-PLA2, lipoprotein-associated phospholipase A2; NfL, neurofilament light chain; NSE, neuron-specific enolase; Oxidized a-Syn, Oxidized a-Synuclein; PK^res^ a-Syn, proteinase-K resistant a-Synuclein; pS-α-syn-RBC, Serine 129-phosphorylated alpha-synuclein red blood cell; PSer129, phospho-Serine 129 a-Synuclein; p-tau181, Phospho-tau 181; SAA, serum amylase A; sdLDL, small dense low-density lipoprotein; SIRT1, silent information regulator 1; SOD, superoxide dismutase; TNF-α, tumor necrosis factor alpha; Total (methanol) a-Syn, Total (methanol) a-Synuclein; Total (cyclohexene) a-Syn, Total (cyclohexene) a-Synuclein; T-tau, total tau; Trf, Transferrin; TTR, transthyretin; VCAM-1, soluble vascular cell adhesion; wrCRP, wide-range C-reactive protein.

#### 3.3.1 Metabolic function

Cys C increased and high-density lipoprotein (HDL) decreased in PD-CI, while most markers showed no consistent changes. These findings suggest metabolic dysregulation in PD-CI, though evidence is limited.

#### 3.3.2 Neuronal function

NfL and *α*-synuclein were elevated in PD-CI, reflecting neuronal damage and protein aggregation, whereas GDNF decreased. NfL and α-synuclein hold promise as diagnostic markers, pending assay standardization.

#### 3.3.3 Inflammatory and immune functions

C-reactive protein (CRP) and IL-6 increased in PD-CI, indicating neuroinflammation, while interleukin-8 (IL-8) decreased. CRP and IL-6 may aid in tracking progression, though other markers lack consistency.

#### 3.3.4 Blood and vascular functions

Vascular cell adhesion molecule-1 (VCAM-1) and fibrinogen rose in PD-CI, hinting at vascular involvement, but most markers showed no clear association. Further research is warranted.

#### 3.3.5 Others

The cell cycle regulator p21 decreased in PD-CI, while other markers were inconsistent, limiting their current utility.

### 3.4 Results of NMA

#### 3.4.1 Network geometry plot

**Figure 2:**
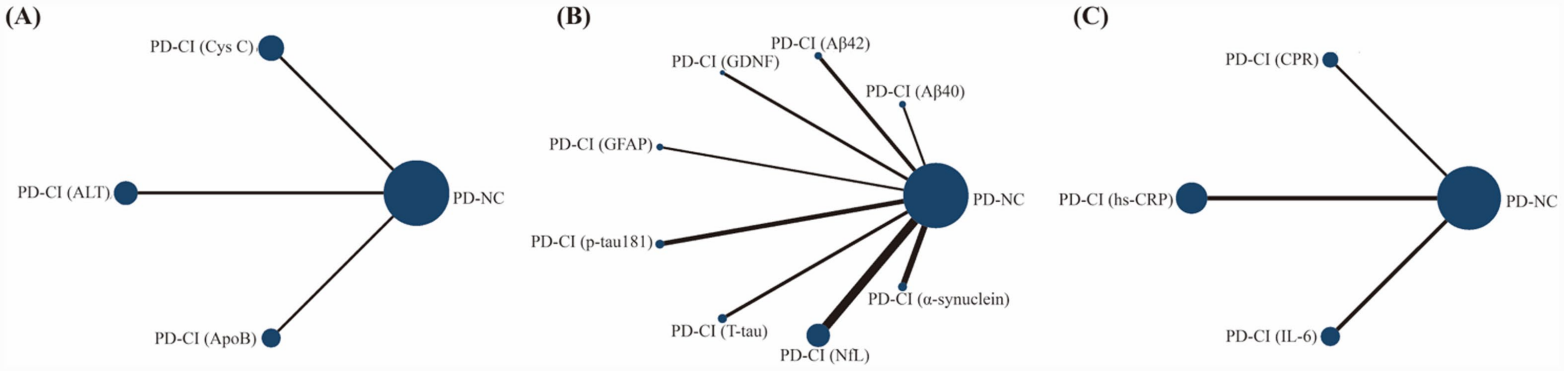
The network geometry plot, **(A)** metabolic-function biomarkers; **(B)** represents neuronal-function biomarkers; **(C)** inflammatory/immune and vascular-function biomarkers.

The network geometry plot (Figure 2) illustrated the available direct comparisons for biomarkers included in the quantitative synthesis. The size of the nodes corresponds to the total sample size for each biomarker, while the thickness of the connecting lines represents the number of studies contributing to each direct comparison. The NfL network had the largest number of studies and participants, providing the most robust evidence.

#### 3.4.2 Quantitative synthesis of key blood-based biomarkers

**Figure 3:**
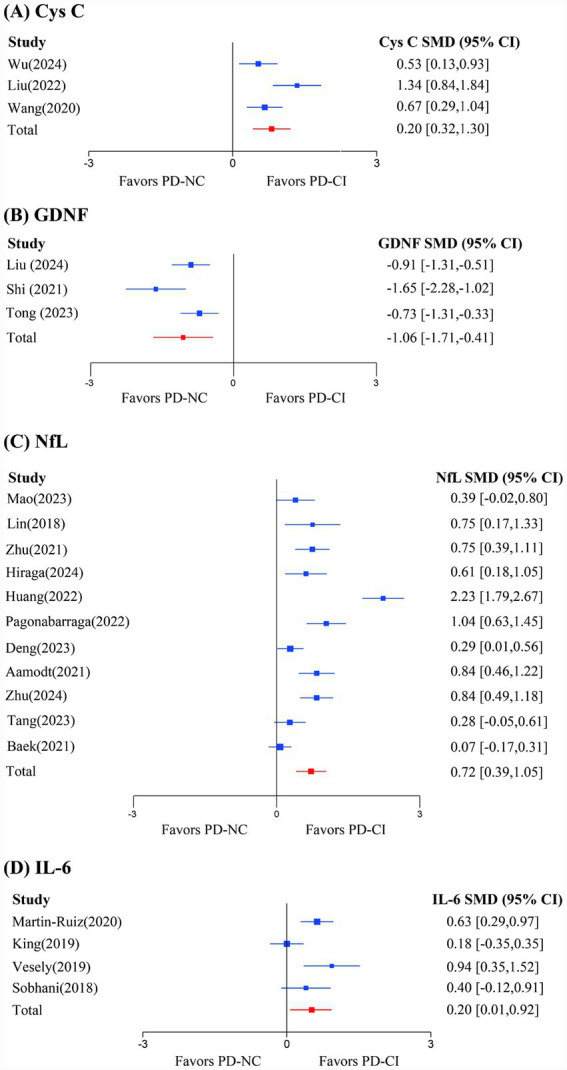
Forest plots of key associated biomarkers.

To synthesize evidence on blood-based protein biomarkers associated with cognitive dysfunction in Parkinson’s disease, we conducted random-effects NMAs for biomarkers reported in at least three studies. In the main text, we highlight four pre-specified key biomarkers (Cys C, GDNF, NfL, and IL-6) because they were consistently reported and showed clinically relevant signals across studies. The corresponding forest plots comparing PD-CI with PD-NC are presented in Figure 3, and we report the number of studies, total participants, pooled SMD with 95% CI, and statistical significance for each marker. Results for additional biomarkers meeting the quantitative synthesis threshold are provided in the Supplementary Figures S1–S3, including metabolic, neuronal, and inflammatory/immune biomarkers that did not show statistically significant differences between PD-CI and PD-NC in the pooled analyses.

##### 3.4.2.1 Cys C

The NMA of Cys C levels included 4 studies with a total of 519 participants. The forest plot (Figure 3A) revealed that Cys C levels were significantly higher in PD-CI compared to PD-NC, with an overall SMD of 0.81 (95% CI: 0.32 to 1.3, *p* = 0.007). This finding suggests that elevated Cys C levels are associated with cognitive impairment in PD, supporting its potential role as a biomarker of metabolic dysfunction in this context.

##### 3.4.2.2 GDNF

The NMA of GDNF levels included 3 studies with a total of 263 participants. The forest plot (Figure 3B) demonstrated significantly lower GDNF levels in PD-CI compared to PD-NC, with an overall SMD of −1.06 (95% CI: −1.71 to −0.41, *p* = 0.002). This reduction in GDNF, a neurotrophic factor, supports its potential protective role against cognitive decline in PD. However, because this analysis included only three studies (total *n* = 263), the precision of the pooled estimate is limited, and the result should be interpreted with caution.

##### 3.4.2.3 NfL

The NMA of NfL levels included 11 studies with a total of 1,647 participants. The forest plot (Figure 3C) demonstrated significantly elevated NfL levels in PD-CI compared to PD-NC, with an overall SMD of 0.72 (95% CI: 0.39 to 1.05, *p* < 0.001). This increase supports NfL’s role as a marker of neuronal damage in the context of PD-related cognitive impairment.

##### 3.4.2.4 IL-6

The NMA of IL-6 levels included 4 studies with a total of 430 participants. The forest plot (Figure 3D) indicated significantly higher IL-6 levels in PD-CI compared to PD-NC, with an overall SMD of 0.20 (95% CI: 0.01 to 0.92, *p* = 0.048). This elevation highlights the association between neuroinflammation and cognitive dysfunction in PD.

#### 3.4.3 Biomarkers with non-significant associations

In addition to the key biomarkers described above, our NMA evaluated several other blood-based protein biomarkers that were reported in three or more studies. No statistically significant differences in circulating levels were found between PD-CI and PD-NC groups for these markers, as detailed in the supplementary forest plots (Supplementary Figures S1–S3). Notably, these non-significant findings were distributed across our pre-specified functional categories, providing a more nuanced view of the biomarker landscape in PD-CI. Within the metabolic function category, biomarkers including alanine transaminase (ALT) and apolipoprotein B (ApoB) did not show significant associations with cognitive status in PD (Supplementary Figure S1). Among neuronal function biomarkers, well-established candidates such as amyloid-*β* (Aβ40, Aβ42), glial fibrillary acidic protein (GFAP), phosphorylated tau (p-tau181), total tau (T-tau), and *α*-synuclein did not demonstrate consistent or significant alterations in PD-CI compared to PD-NC (Supplementary Figure S2). This suggests that, despite their central role in PD and other neurodegenerative pathologies, their utility as discriminative blood-based biomarkers for PD-CI may be limited relative to NfL and GDNF within the same functional category for inflammatory and immune markers, both CRP and high-sensitivity C-reactive protein (hs-CRP) overall results were not statistically significant, despite trends observed in individual studies (Supplementary Figure S3). The lack of a robust signal for these general inflammatory markers, in contrast to the specific cytokine IL-6, hints at the potential importance of measuring more specific inflammatory pathways rather than broad systemic inflammation in the context of PD-CI.

## 4 Discussion

This systematic review and NMA of 47 studies provide robust evidence that blood-based protein biomarkers—Cys C, GDNF, NfL and IL-6—are significantly associated with cognitive dysfunction in Parkinson’s disease. These biomarkers reflect distinct pathological processes: metabolic dysfunction (Cys C), reduced neuroprotection (GDNF), neuronal injury (NfL) and neuroinflammation (IL-6). By classifying biomarkers into five functional categories (metabolic, neuronal, inflammatory, vascular, and others), this study establishes a structured framework to elucidate their roles in PD-related cognitive impairment, advancing the potential for early diagnosis and targeted therapeutic interventions.

### 4.1 Cys C and metabolic dysregulation

Elevated Cys C levels in PD-CI (SMD = 0.81) suggest a role for metabolic dysregulation in cognitive decline. Our findings critically refine this broad concept by demonstrating a clear hierarchy of informativeness among metabolic markers. While Cys C showed a significant association with PD-CI, other markers reflecting general hepatic function (ALT, AST) or peripheral lipid metabolism (ApoB) did not exhibit statistically significant differences between PD-CI and PD-NC groups. This observed dissociation suggests that Cys C may capture aspects of metabolic dysregulation distinct from those reflected by these other markers, potentially indicating specific pathological processes relevant to PD.

The clinical interpretation of Cys C is complex due to its well-known role as a marker of renal function (Zhang et al., 2022). Its association with PD-CI in our analysis, in contrast to the non-significant findings for other routine metabolic measures, suggests a potential signal beyond general systemic health. Nonetheless, this observation is based on unadjusted comparisons, and future studies should evaluate Cys C’s role while controlling for confounders like renal function to clarify its specificity in PD-CI. Cys C, a cysteine protease inhibitor, is primarily a marker of kidney function but may also reflect broader metabolic disturbances or inflammation in neurodegenerative diseases (Imarisio et al., 2021). Koníčková et al. (2023) identified elevated levels in alpha-synucleinopathies, including PD, corroborating our findings. However, the variability in Cys C results echoes findings in Alzheimer’s disease, where its role remains debated (Nair et al., 2020). The significant association of Cys C with cognitive impairment suggests it could contribute to a multi-biomarker panel for PD-CI, although its precise role in PD pathology requires further investigation due to potential influences from systemic factors such as kidney function (Liu et al., 2025).

The clinical utility of Cys C is limited by its lack of specificity, as it is influenced by renal function and systemic inflammation, which are common in aging populations and PD patients (Lertnawapan et al., 2012). Nevertheless, its elevation in PD-CI may reflect broader metabolic disturbances, such as lysosomal dysfunction or impaired protein clearance, which are implicated in PD pathogenesis (Lee et al., 2007). For instance, Mi et al. demonstrated that Cys C enhances amyloid-beta pathology in AD models, suggesting a similar role in PD-related proteinopathies (Mi et al., 2009). Future research should explore Cys C’s interactions with *α*-synuclein aggregation and other metabolic pathways in PD, as well as its specificity for cognitive impairment compared to other systemic conditions.

### 4.2 GDNF and neuroprotection

The significant reduction in GDNF levels (SMD = −1.06) strongly supports the role of impaired neurotrophic support in PD-CI. Notably, among the four key biomarkers, GDNF showed the largest absolute SMD, indicating the strongest standardized separation between PD-CI and PD-NC in our synthesis. However, the magnitude of an SMD is a unitless measure of between-group difference and does not in itself establish causality or imply that GDNF is a core intervention target. This finding gains further weight when considered alongside other neurotrophic or protective factors within our framework that showed no significant association. For instance, factors like α-Klotho (Yalcin et al., 2025) and SIRT1 (Scheper et al., 2023), which have broad anti-aging and neuroprotective roles, did not demonstrate consistent alterations in our analysis. GDNF is crucial for the survival and function of dopaminergic neurons, and its deficiency is implicated in both motor and non-motor symptoms of PD (Lindholm and Saarma, 2022). The association between GDNF and cognitive impairment is supported by Tong et al. (2023), who found that lower serum GDNF levels are linked to executive dysfunction in PD. Tang et al. (2024) further demonstrated that GDNF, combined with imaging parameters, predicts cognitive status, reinforcing its biomarker potential. The substantial effect size of GDNF underscores its potential as a biomarker of neurotrophic deficits in PD, reflecting the loss of neuroprotective mechanisms that sustain cognitive function.

From a translational perspective, GDNF has been actively explored as a disease-modifying strategy; however, clinical studies of intracerebral delivery of GDNF (and related GDNF-family factors) have reported mixed outcomes, with randomized trials not meeting their primary clinical endpoints (Barker et al., 2020). In addition, our GDNF estimate is based on only three studies, with heterogeneous assay methods; therefore, despite the large SMD, this finding should be regarded as preliminary and requires confirmation in larger, multi-center cohorts with standardized measurement and cognitive outcomes. Importantly, cognitive outcomes have been limited in prior interventional studies, and therefore current evidence is insufficient to define GDNF as an established ‘core intervention target’ for PD-related cognitive impairment (Bao et al., 2020; Behl et al., 2020). However, the delivery of GDNF to the brain remains a challenge due to its limited ability to cross the blood–brain barrier, necessitating advanced delivery systems like viral vectors or convection-enhanced delivery (Wang et al., 2022). Additionally, the variability in GDNF measurement methods across studies highlights the need for standardized assays to ensure reliable results. Future research should focus on longitudinal studies to assess GDNF’s predictive value for cognitive decline, its interactions with other biomarkers, and the efficacy of GDNF-based therapies in preserving cognitive function in PD. Accordingly, we describe GDNF as a promising candidate pathway/target that should be prioritized for future mechanistic and neuroprotective trials with cognitive endpoints.

### 4.3 NfL and neuronal injury

The significant elevation of NfL in PD patients with cognitive impairment compared to those without (SMD = 0.72) underscores its role as a marker of axonal damage and neurodegeneration, a hallmark of PD and other neurodegenerative disorders (Aamodt et al., 2021; Frigerio et al., 2023). This finding is further supported by its contrast with the non-significant results for other well-established neuronal proteins in our analysis. Notably, amyloid-*β* (Gupta and Goyal, 2016), T-tau (Marks et al., 2021), p-tau181 (Telser et al., 2023), and total *α*-synuclein (Gonzalez et al., 2019)—despite their undeniable centrality to neurodegenerative pathology—did not show consistent diagnostic utility in blood. This dichotomy is consistent with our NMA results, in which Aβ40, Aβ42, p-tau181, total tau, and total α-synuclein did not show statistically significant differences between PD-CI and PD-NC. Therefore, rather than implying superiority of one pathology over another, our findings suggest a practical consideration for blood-based biomarker discovery in PD-CI; biomarkers that directly reflect active neuronal injury, such as NfL, may provide a stronger and more reproducible peripheral signal in blood within the current evidence base (Gaetani et al., 2019; Pagonabarraga et al., 2022). In contrast, circulating measures of aggregation related proteins, including Aβ, tau, and α-synuclein, may be less discriminative in blood, which may partly reflect low concentrations, assay and methodological heterogeneity, and potential confounding from peripheral sources (Chiu et al., 2021).

This interpretation is reinforced by the biology of NfL. As a structural component of axons, NfL is released upon neuronal injury, making it a direct and sensitive indicator of ongoing neurodegeneration (Gaetani et al., 2019). Our findings align with prior studies linking elevated NfL to cognitive decline in PD (Batzu et al., 2022) and its progression across neurodegenerative diseases (Gotze et al., 2024). While NfL’s specificity for PD cognitive impairment is limited, as it is elevated in other conditions like AD (Sanchez et al., 2024), its robust effect size and reliability across studies position it as a cornerstone biomarker for quantifying neurodegenerative activity within a multi-modal diagnostic framework.

NfL’s clinical utility is therefore twofold. First, its ability to detect neuronal damage potentially before overt cognitive symptoms appear makes it a valuable tool for risk stratification and routine monitoring. Second, its correlation with disease progression and response to neuroprotective interventions in preclinical models (Lisi et al., 2025) suggests strong potential as a pharmacodynamic biomarker for clinical trials (Pedersen et al., 2024). The primary challenges moving forward are the standardization of assays and the strategic integration of NfL with other biomarkers to enhance diagnostic specificity for PD-CI beyond general neurodegeneration. Future longitudinal studies should focus on defining its predictive value and refining its role within a multi-biomarker panel.

### 4.4 IL-6 and neuroinflammation

The significant elevation of IL-6 in PD-CI (SMD = 0.20), albeit modest, highlights a critical distinction within the inflammatory landscape of PD. Our findings suggest a hierarchy of specificity among inflammatory markers. IL-6, a pro-inflammatory cytokine, crosses the blood–brain barrier and activates microglia and astrocytes, triggering the release of additional inflammatory mediators such as tumor necrosis factor-alpha (TNF-α) and interleukin-1 beta (IL-1β) (Fellner et al., 2013; Ni et al., 2019). These mediators can exacerbate neuronal damage and disrupt neural networks critical for cognitive functions, including executive function, memory, and visuospatial abilities (Menza et al., 2010). This direct role in neuro-immune crosstalk may explain why IL-6, as a specific inflammatory mediator, showed a significant association, whereas broader, downstream markers of systemic inflammation like CRP and hs-CRP did not. CRP, primarily produced by the liver in response to IL-6, is a robust but non-specific marker of systemic inflammation (Li et al., 2024). However, this contrast should not be overinterpreted as a ‘dissociation’ or definitive evidence of distinct pathological pathways, because statistical significance is influenced by effect size, uncertainty, and between-study heterogeneity. Given the modest effect size of IL-6 and the uncertainty of the estimates, the apparent difference between IL-6 and CRP/hs-CRP should be considered exploratory and requires confirmation in well-controlled, head-to-head studies reporting comparable effect sizes and confidence/credible intervals.

Our results are consistent with prior research demonstrating elevated IL-6 levels in the CSF and serum of PD patients, correlating with disease severity and progression (Hofmann et al., 2009; Vesely et al., 2019). For instance, Hofmann KW reported increased IL-6 in the CSF of *de novo* PD patients, while Brodacki et al. found a significant association between peripheral IL-6 levels and PD pathology (Brodacki et al., 2008; Hofmann et al., 2009). Additionally, genetic polymorphisms in the IL-6 gene have been linked to increased PD susceptibility, suggesting a genetic basis for IL-6’s role in disease progression (Hakansson et al., 2005).

Despite the statistical significance, the modest effect size of IL-6 (SMD = 0.20, 95% CI: 0.01 to 0.92, *p* = 0.048) indicates that it may not be a standalone biomarker for PD cognitive impairment. However, its specific role in neuroinflammation makes it a promising component of a multi-biomarker panel. Preclinical studies have shown that IL-6 inhibition, such as with Tocilizumab, an anti-IL-6 receptor antibody, can mitigate neuroinflammation and protect neurons in PD models, suggesting potential therapeutic applications (Pons-Espinal et al., 2024). Furthermore, IL-6’s association with cognitive decline in other neurodegenerative diseases, such as Alzheimer’s disease, where it contributes to synaptic dysfunction, supports its relevance in PD (Jarne-Ferrer et al., 2022). Future research should explore IL-6’s interactions with other inflammatory markers and its longitudinal dynamics to better understand its contribution to cognitive impairment.

### 4.5 Clinical implications

Finding Cys C, GDNF, NfL, and IL-6 as biomarkers for cognitive impairment related to PD has important clinical effects. Because they are non-invasive, they can be used for routine screening. This could help find cognitive decline earlier than current neuropsychological tests or invasive CSF tests. NfL’s strong link to neuronal injury makes it a top choice for figuring out who is most at risk for PD-CI. IL-6, although its effect size is small, may improve diagnostic accuracy when used with other biomarkers that show the inflammatory part of PD pathology. The decrease in GDNF underscores the promise of neurotrophic therapies, whereas Cystatin C may elucidate metabolic factors influencing cognitive decline. A multi-biomarker panel that includes these markers could give a full picture of cognitive status by measuring different pathological processes, such as neurodegeneration, inflammation, neuroprotection, and metabolic dysfunction.

### 4.6 Strengths and limitations

This study has several strengths, including a comprehensive search across five databases, explicit eligibility criteria with duplicate screening and extraction, and a pre-specified frequentist synthesis with study quality assessment using the Newcastle–Ottawa Scale. In addition, classifying biomarkers by functional domain provides a biologically informed framework to interpret heterogeneous candidates. Several limitations merit consideration. Between-study heterogeneity is likely due to differences in participant characteristics, cognitive definitions, and assay methodologies, and some biomarkers were informed by few studies, limiting precision. Most included studies were cross-sectional, precluding inference on temporality or prognostic value. Moreover, the evidence network was predominantly star-shaped with limited head-to-head evidence; therefore, biomarker-to-biomarker comparisons or ranking approaches are not supported as confirmatory and should be considered, at most, hypothesis-generating. Finally, harmonizing cognitive status may introduce residual misclassification. Accordingly, the pooled estimates should be interpreted with caution, and future work should prioritize standardized cognitive criteria, harmonized assays, and longitudinal head-to-head cohorts.

### 4.7 Future research directions

Future research should focus on improving methodological standardization and strengthening clinical translation. First, harmonized protocols for biomarker measurement and for cognitive phenotyping are needed to enhance comparability across studies. Second, adequately powered longitudinal cohorts should evaluate within-person trajectories of biomarker change and determine prognostic utility for incident PD-MCI/PDD and cognitive decline. Third, head-to-head studies measuring multiple biomarkers within the same individuals should report clinically interpretable performance metrics, including discrimination, calibration, and decision-analytic measures, to assess whether multi-biomarker panels provide incremental value beyond single markers. Where feasible, individual participant data meta-analyses would enable multivariable adjustment and mediation modelling to clarify the independence of associations and to quantify the added value of combinations such as NfL with metabolic or inflammatory markers. Fourth, mechanistic studies integrating biofluids with neuroimaging and neuropathological correlates are warranted to elucidate biological pathways linking these biomarkers to PD-related cognitive impairment and to identify tractable therapeutic targets. Finally, future cohorts should include diverse populations to improve generalizability, and interventional studies targeting IL-6- and GDNF-related pathways should incorporate cognitive endpoints to evaluate potential disease-modifying effects.

## 5 Conclusion

This NMA establishes Cys C, GDNF, NfL and IL-6 as promising blood-based biomarkers for cognitive impairment in PD, reflecting neuronal injury, neuroinflammation, reduced neuroprotection, and metabolic dysregulation. Their integration into a multi-biomarker panel holds significant potential for early diagnosis, risk stratification, and personalized treatment strategies. Further research is needed to standardize measurement techniques, validate multi-biomarker approaches, and explore therapeutic interventions targeting these biomarkers to improve cognitive outcomes in PD.

## Data Availability

The original contributions presented in the study are included in the article/Supplementary material, further inquiries can be directed to the corresponding authors.
