# Supplementary material for: Potential protein blood-based biomarkers for cognitive dysfunction in Parkinson’s disease: a systematic review and network meta-analysis
Source: Front Aging Neurosci. 2026 Feb 10;18:1724548. doi: 10.3389/fnagi.2026.1724548 (PMC12929444; doi:10.3389/fnagi.2026.1724548)
Supplement: Supplementary file 1 [file Data_Sheet_1.pdf]

Supplementary Material

Supplementary table 1. Search strategy		
**1A: Search Strategy for PubMed**		
**Search Number**	**Search Terms**	**Search Logic**
#1	"Parkinson disease"[MeSH Terms] OR "parkinsonian disorders"[MeSH Terms] OR Parkinson*[Title/Abstract] OR PD[Title/Abstract]	Parkinson's Disease-related terms
#2	"cognition disorders"[MeSH Terms] OR cognitive*[Title/Abstract] OR dementia[MeSH Terms] OR dementia* [Title/Abstract]	Cognitive disorders-related terms
#3	Biomarkers[MeSH Terms] OR biomarker* [Title/Abstract] OR marker* [Title/Abstract]	Biomarkers-related terms
#4	blood[Title/Abstract] OR serum[Title/Abstract] OR plasma[Title/Abstract]	Sample type-related terms
#5	#1 AND #2 AND #3 AND #4	Combined search query
**1B: Search Strategy for EMBASE**		
#1	'parkinson disease'/exp OR 'parkinsonian disorders'/exp OR parkinson*:ab ,ti OR PD: ab ,ti	Parkinson's Disease and related terms using MeSH and text search
#2	'cognition disorders'/exp OR cogniti*:ab,ti OR dementia/exp OR dementia*:ab,ti	Cognitive disorders and related terms using MeSH and text search
#3	biomarkers/exp OR biomarker*:ab,ti OR marker*:ab,ti	Biomarkers and related terms using MeSH and text search
#4	blood:ab,ti OR serum:ab,ti OR plasma:ab,ti	Sample types: blood, serum, plasma (title/abstract search)
#5	#1 AND #2 AND #3 AND #4	Combined search query for all conditions and sample types
**1C: Search Strategy for web of science**		
#1	TS=("parkinson disease" OR "parkinsonian disorders" OR parkinson* OR PD)	Search for Parkinson's Disease-related terms in the topic (TS) field
#2	TS=("cognition disorders" OR cogniti* OR dementia*)	Search for cognitive disorders and dementia-related terms in the topic (TS) field
#3	TS=(biomarkers OR biomarker* OR marker*)	Search for biomarkers and related terms in the topic (TS) field
#4	TS=(blood OR serum OR plasma)	Search for sample types (blood, serum, plasma) in the topic (TS) field
#5	#1 AND #2 AND #3 AND #4	Combined search query for all conditions and sample types
**Index**: SCI-EXPANDED, SSCI, CPCI-S, CPCI-SSH, ESCI		
**1D: Search Strategy for Cochrane Library**		
#1	[mh "parkinson disease"]	MeSH term for Parkinson’s Disease
#2	[mh "parkinsonian disorders"]	MeSH term for Parkinsonian Disorders
#3	(parkinson*):ti, ab,kw	Search in title, abstract, and keywords for Parkinson-related terms
#4	(PD): ti, ab,kw	Search in title, abstract, and keywords for PD (Parkinson's Disease)

#5	#1 OR #2 OR #3 OR #4	Combined search query for Parkinson’s Disease-related terms
#6	[mh "cognition disorders"]	MeSH term for Cognition Disorders
#7	(cogniti*): ti, ab,kw	Search in title, abstract, and keywords for cognitive disorders
#8	[mh dementia]	MeSH term for Dementia
#9	(dementia*): ti, ab,kw	Search in title, abstract, and keywords for dementia-related terms
#10	#6 OR #7 OR #8 OR #9	Combined search query for cognitive disorders
#11	[mh biomarkers]	MeSH term for Biomarkers
#12	(biomarker*): ti, ab,kw	Search in title, abstract, and keywords for biomarkers
#13	(marker*):ti,ab,kw	Search in title, abstract, and keywords for markers
#14	#11 OR #12 OR #13	Combined search query for biomarkers
#15	(blood): ti, ab, kw	Search in title, abstract, and keywords for blood
#16	(plasma): ti, ab, kw	Search in title, abstract, and keywords for plasma
#17	(serum): ti, ab, kw	Search in title, abstract, and keywords for serum
#18	#15 OR #16 OR #17	Combined search query for blood, plasma, or serum
#19	#5 AND #10 AND #14 AND #18	Final combined search query for Parkinson’s disease, cognition disorders, biomarkers, and sample types (blood, plasma, serum)
**1E: Search Strategy for Scopus**		
#1	TITLE-ABS-KEY ("parkinson disease" OR "parkinsonian disorders" OR parkinson* OR PD)	Search for Parkinson’s Disease-related terms in title, abstract, and keywords (TITLE-ABS-KEY)
#2	TITLE-ABS-KEY ("cognition disorders" OR cogniti* OR dementia*)	Search for cognitive disorders and dementia-related terms in title, abstract, and keywords (TITLE-ABS-KEY)
#3	TITLE-ABS-KEY (biomarkers OR biomarker* OR marker*)	Search for biomarkers and related terms in title, abstract, and keywords (TITLE-ABS-KEY)
#4	TITLE-ABS-KEY (blood OR serum OR plasma)	Search for sample types (blood, serum, plasma) in title, abstract, and keywords (TITLE-ABS-KEY)
#5	#1 AND #2 AND #3 AND #4	Final combined search query for Parkinson’s Disease, cognition disorders, biomarkers, and sample types (blood, serum, plasma)

Note: Search time range for all tables is from inception to 23 January 2025.

Supplementary Table 2. Quality assessment.											
**No.**	**Author**	**Year**	**Selection**				**Comparability**	**Exposure**			**Total**
			**1**	**2**	**3**	**4**	**1**	**1**	**2**	**3**	
1	Zubo Wu	2024		★	★	★	★	★	★	★	7
2	Suaad Abd Elhadi	2019		★	★	★	★	★	★	★	7
3	Chao Hou	2024	★	★	★	★	★★	★	★	★	9
4	Cheng-Hsuan Li	2022			★	★	★	★	★	★	6
5	Xu-Ying Li	2021	★	★	★	★	★★	★	★	★	8
6	Shuai Mao	2023			★	★	★★	★	★		6
7	Hsin-Hsi Tsai	2021			★	★	★	★	★	★	6
8	In-Uk Song	2013	★		★	★	★★	★	★	★	8
9	Yung-Shuan Lin	2018			★	★	★	★	★	★	6
10	Mei-Xue Dong	2021	★		★	★	★	★	★	★	7
11	Hailing Liu	2022		★	★	★	★	★	★	★	7
12	Mei-XueDong	2017	★	★	★	★	★	★	★	★	8
13	Yi Liu	2024	★		★	★	★	★	★	★	7
14	Kuiper, M. A	1993			★	★	★	★	★	★	6
15	Ting Wang	2020		★	★	★	★★	★	★	★	8
16	Yongyun Zhu	2021	★		★		★	★	★	★	6
17	Gerard J. van Kamp	1995	★		★	★	★	★	★	★	7
18	Qianqian He	2024	★		★		★★	★	★	★	7
19	Dawei Fan	2020			★	★	★	★	★	★	6
20	Karin Gmitterova	2019			★	★	★	★	★	★	6
21	Chin-Hsien Lin	2021			★	★	★	★	★	★	6
22	Chun-Chao Huang	2024	★	★	★	★	★★	★	★	★	9
23	Caranci, G	2013	★		★	★	★★	★	★		7
24	Keita Hiraga	2024			★	★	★	★	★	★	6
25	Carmen Martin-[illegible]	2020	★	★	★	★	★	★	★	★	8
26	Ahmet Yalcin	2025		★	★	★	★	★	★	★	7
27	Walter Maetzler	2012			★	★	★	★	★	★	6
28	Yixian Huang	2022			★	★	★	★	★	★	6
29	Javier [illegible]	2022	★	★	★	★	★★	★	★	★	9
30	Xiao Deng	2023		★	★	★	★★	★	★	★	8

31	Whitley W	2021			★	★	★★	★	★	★	7
32	Nor A. Samat	2017		★	★	★	★	★	★	★	7
33	King, E	2019		★	★	★	★	★	★	★	7
35	Elena Contaldi	2022			★	★	★	★	★	★	6
36	Yongyun Zhu	2024			★	★	★	★	★	★	6
37	Yilin Tang	2023		★	★	★	★	★	★	★	7
38	Chin-Hsien Lin	2019	★	★	★		★	★	★	★	7
39	Ming-Yu Shi	2021		★	★	★	★★	★	★	★	8
40	Yuting Zhu	2020		★	★		★	★	★	★	6
41	Seong-Min Choi	2016		★	★	★	★★	★	★	★	8
42	Shu-Yan Tong	2023		★	★	★	★★	★	★	★	8
43	Mi Xiong	2022	★		★	★	★	★	★	★	7
44	Keke Liang	2024	★	★	★	★	★★	★	★	★	9
45	Chiu, M. J	2021			★	★	★★	★	★	★	7
46	Min Seok Baek	2021	★	★	★	★	★★	★	★	★	9
47	Branislav Veselý	2019		★	★	★	★★	★	★	★	8
48	Ahmad Sobhani	2018	★	★	★	★	★	★	★	★	8

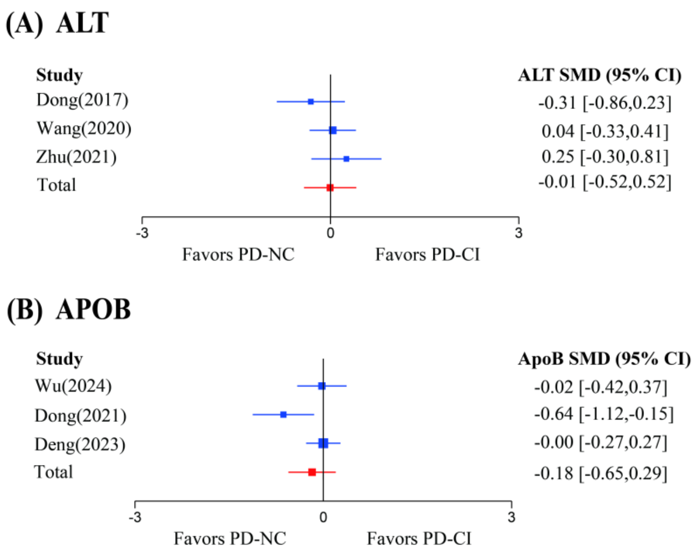

Supplementary Figure 1. Forest plots of metabolic biomarkers

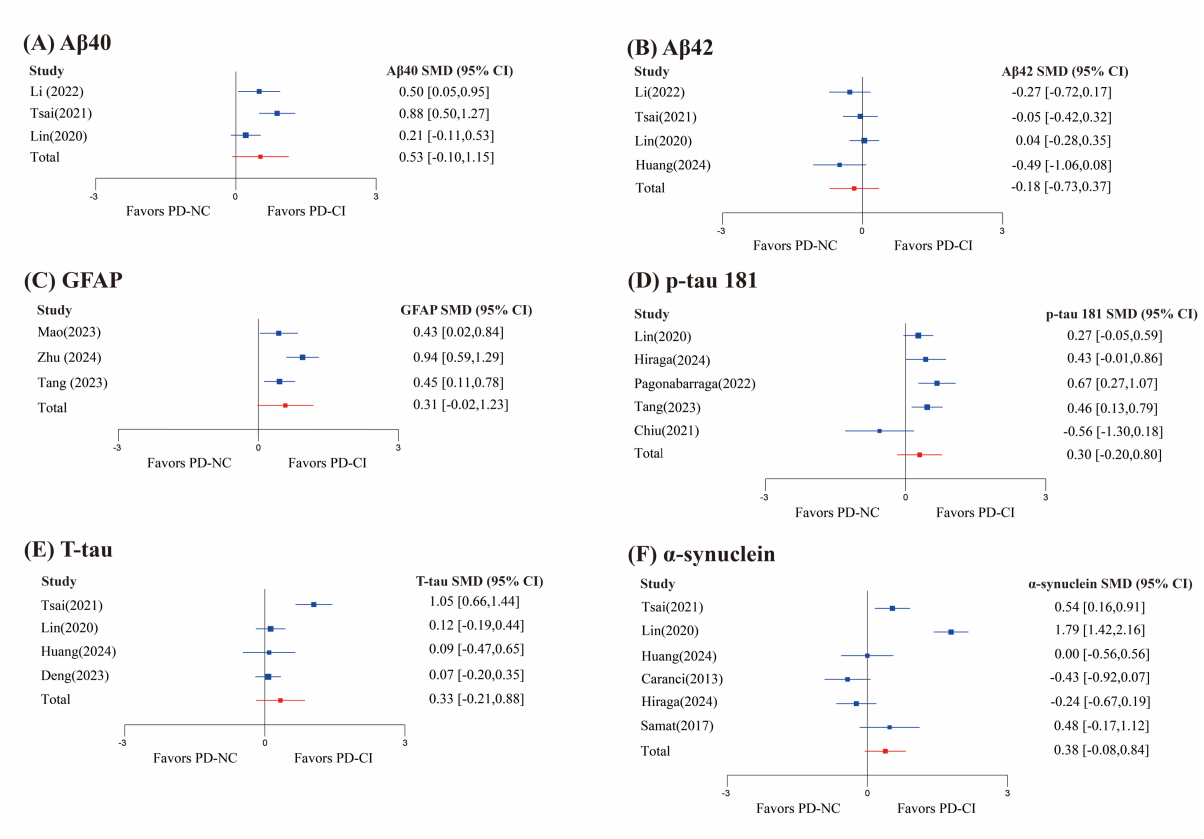

Supplementary Figure 2. Forest plots of neuronal biomarkers

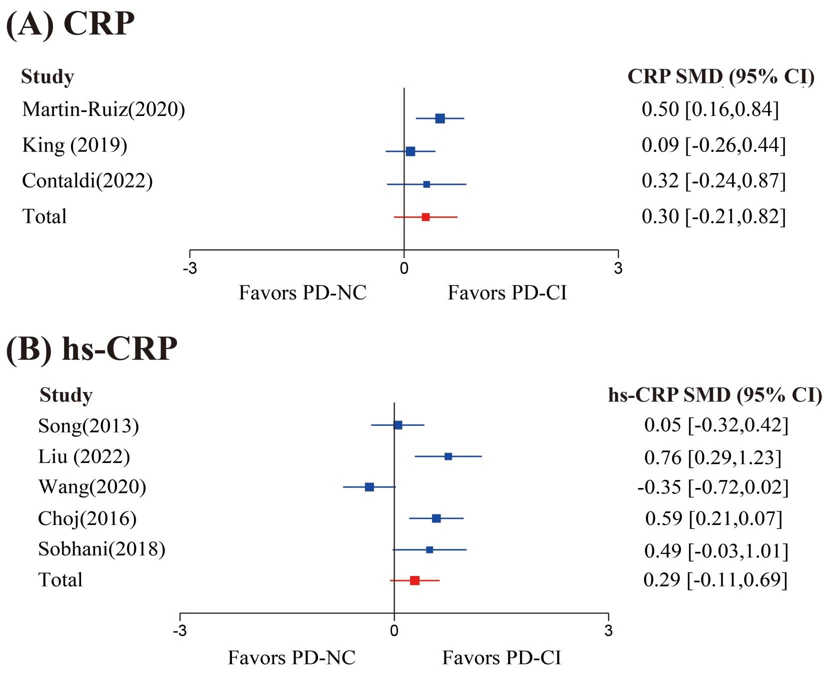

Supplementary Figure 3. Forest plots of inflammatory biomarkers
